# Extended treatment of abrocitinib: evaluation of efficacy and safety in chronic actinic dermatitis

**DOI:** 10.3389/fmed.2026.1742273

**Published:** 2026-02-04

**Authors:** Lu Tang, Lanhai Zhong, Liangzi Liang, Jiande Han, Naiyu Lin

**Affiliations:** Department of Dermatology, The First Affiliated Hospital, Sun Yat-sen University, Guangzhou, China

**Keywords:** abrocitinib, chronic actinic dermatitis, efficacy, safety, differentially expressed proteins

## Abstract

**Introduction:**

JAK inhibitors are well-established for their utilization in the treatment of autoinflammatory diseases, with the JAK1 inhibitor abrocitinib primarily employed in the management of atopic dermatitis. To provide new clinical therapeutic experience for patients with chronic actinic dermatitis (CAD), reveal the inflammatory pathways that may be involved in the treatment of CAD with abrocitinib and to provide clues for exploring the pathogenesis of CAD.

**Methods:**

In a 3-month longitudinal study, we recorded and analyzed laboratory test data and clinical severity assessments in 16 patients diagnosed with chronic actinic dermatitis treated with abrocitinib. And the plasma samples of 6 patients before and after treatment were analyzed proteomically using Olink inflammation panel.

**Results:**

After 12 weeks of treatment, there was a significant decrease in disease severity scores of 16 patients. No serious adverse events were identified throughout the course of treatment. Significant changes in several inflammatory factors such as EN-RAGE, MCP-3, MCP-4, IL-13, CCL4, FGF21 and TNFRSF9 were observed after treatment.

**Discussion:**

The analysis of our data demonstrates that abrocitinib may be an effective therapeutic option in the management of CAD. IL-13 and CCL-4 might be the core effective factors after the therapy.

## 1 Introduction

Chronic actinic dermatitis (CAD) is a chronic skin condition, often resulting from prolonged exposure to sunlight (1). CAD typically presents as a persistent and challenging condition, characterized by year-round severe pruritus. Furthermore, it predominantly affects elderly males, with some patients having underlying comorbidities that further complicate the choice of suitable treatment options.

Previous research has illuminated certain parallels between CAD and atopic dermatitis (AD) in terms of clinical manifestations, Th2 polarization, and skin barrier disruption (2, 3). These similarities suggest shared aspects in their pathogenesis, as well as overlapping strategies in drug treatments. Abrocitinib, a selective JAK1 inhibitor, has demonstrated efficacy in the management of moderate-to-severe AD (4). Previous literature has also reported favorable outcomes with JAK inhibitors, like baricitinib and tofacitinib, in cases of refractory CAD (5, 6). Our study was therefore designed to offer clinical insights into the use of abrocitinib for refractory CAD, systematically evaluating its efficacy and safety profile in managing this challenging condition. We also conducted an in-depth investigation of inflammatory protein changes in CAD patients before and after abrocitinib treatment at the proteomic level, providing insights into therapeutic mechanisms and contributing to a deeper understanding of CAD pathogenesis.

## 2 Materials and methods

### 2.1 Study design, population, and data source

We analyzed the clinical data of CAD patients from the dates of 1 January, 2023, to 30 June, 2024. Patients suffered from CAD satisfied the following criteria (7): (1) chronic eczematoid skin lesions characterized by erythema, infiltrating papules, and plaques, which predominantly affect sun-exposed areas but may also extend to non-exposed areas; (2) the condition persists for more than 3 months, with recurring episodes; (3) reduced minimal erythema dose (MED) indicates sensitivity to UVA and/or UVB; and (4) findings consistent with CAD pathology support the diagnosis. Patient demographics along with details such as the duration of CAD, comorbidities, MED results, and prior treatment history, were systematically collected and analyzed. Following thorough discussions with each patient and obtaining informed consent, the treatment regimen commenced with a daily administration of abrocitinib (100 mg). Throughout the treatment course, patients were permitted to use topical low-potency or moderate-potency corticosteroids, topical calcineurin inhibitors, and oral antihistamines. These adjunct treatments were gradually tapered and eventually discontinued after 4–12 weeks of abrocitinib therapy.

### 2.2 Patient clinical severity assessment

The eczema area and severity index (EASI), the three-level version of the EuroQol five-dimensional (EQ-5D-3L), the investigator’s global assessment (IGA), and the numeric rating scale (NRS)-itch scores were performed at baseline and after 1, 4, and 12 weeks of treatment. The time trade-off values of EQ-5D-3L were calculated by using a model consistent with the characteristics of the Chinese population (8). All ratings were conducted by two independent dermatologists, and the results were then averaged. In cases where there was a significant difference in ratings (more than 5 points), one more unbiased investigator with greater clinical experience was consulted for further evaluation.

We also employed a novel scoring system known as the clinical active score of chronic actinic dermatitis (CAS-CAD) to assess the abrocitinib efficacy. The clinical response of CAD was evaluated on 1, 4, and 12 weeks based on the decrease of the CAS-CAD scores, categorized as excellent (>75% improvement), good (50%–75% improvement), partial (25%–50% improvement), and no response (<25% improvement). This scoring system draws inspiration from the EASI used in AD and the present clinical severity score of chronic actinic dermatitis (CSS-CAD) (9).

In the CAS-CAD scoring process, patients are initially classified into two categories: Type 1 (long pants) and Type 2 (shorts) (Supplementary Figure 1). The distinguishing feature is whether the lower legs are exposed. Subsequently, different weightages are assigned to skin lesions on exposed and non-exposed areas (with exposed areas assigned a weight of 2, and non-exposed areas a weight of 1). The degree of involvement and severity of skin lesions in each category is calculated independently (Supplementary Figure 1).

The final total score is the sum of the following product calculated for each body region: the sum of severity scores for different lesion types (erythema, papules/plaques, scaling, lichenification), multiplied by the affected area score, the proportion of body surface area (BSA), and the region weight. For Type 2 patients, the total score is corrected using a correction coefficient based on the ratio of light-exposed parts in Type 1 and Type 2 (Supplementary Figure 1).

This scoring system confers the benefit of characterizing disease severity based on the extent of involvement of light-exposed and non-light-exposed skin areas while accommodating variations in patients’ clothing preferences. It further underscores the critical relationship between light exposure-induced skin alterations and disease severity.

### 2.3 Identification of differential protein expression

We collected blood samples from six CAD patients at baseline, after 1 week, and after 12 weeks of abrocitinib treatment. Plasma was isolated through centrifugation and stored at −80 °C until further analysis. Proteins were measured using the Olink^®^ target 96 inflammation panel* (Olink Proteomics AB, Uppsala, Sweden), consisting of 92 markers of inflammatory proteins and 4 mid-quality control proteins, according to the manufacturer’s instructions. Data were then quality controlled and normalized using an internal extension control and an inter-plate control to adjust for intra- and inter-run variation.

The final assay read-out is presented in normalized protein expression (NPX) values, an arbitrary log2-scale unit where higher values indicate greater protein expression. A paired-sample *t*-test was used to identify differentially expressed proteins, with a significance threshold of *p* < 0.05. The StringDB database was utilized for interaction analysis, allowing us to construct a protein–protein interaction (PPI) network of the differentially expressed proteins (DEP). Gene ontology (GO) and Kyoto encyclopedia of genes and genomes (KEGG) enrichment analyses were conducted using *ggplot2*.

### 2.4 Ethics statement

The Medical Ethics Committee of the First Affiliated Hospital, Sun Yat-sen University, approved the study protocol and the approval number is No. [2023]007. Informed consent was obtained from the patients for the publication of their case details.

### 2.5 Statistical analysis

All study data were stored in a standard Excel database. All statistical analyses were conducted using IBM SPSS version 25. Continuous variables were expressed as mean (x) ± SD. Continuous data conforming to a normal distribution were analyzed using the paired *t*-test, whereas non-normally distributed continuous data were analyzed using the Wilcoxon signed-rank test. Interrater reliability was assessed using the intraclass correlation coefficient (ICC) through the two-way mixed model. The ICC ranges from 0 to 1, with higher values indicating greater reliability or agreement. ICC values less than 0.5, between 0.5 and 0.75, between 0.75 and 0.9, and greater than 0.9, indicate poor, moderate, good, and excellent reliability, respectively. Results are reported with 95% confidence interval (CI) and a value of *p* < 0.05 was considered statistically significant.

## 3 Results

### 3.1 Efficacy of abrocitinib in treating CAD

We conducted a study encompassing 16 patients with a mean age of 66.50 ± 11.82 years, who were treated off-label with abrocitinib. The average duration of the disease was 4.43 ± 4.41 years. MED results revealed that eight patients (50%) exhibited sensitivity to both UVA and UVB, seven (43.75%) were solely sensitive to UVA, and one (6.25%) only sensitive to UVB (Table 1). All participants had previously received treatments including antihistamines, topical corticosteroids, and/or topical calcineurin inhibitors, yet these approaches yielded unsatisfactory outcomes (Table 2). One patient had previously been treated with dupilumab, but the patient condition deteriorated. Throughout the treatment with abrocitinib, patients continued the use of topical medications or oral antihistamines. These concomitant treatments were gradually tapered and eventually discontinued after 4–12 weeks of abrocitinib therapy.

**Table 1:** Demographic data of CAD patients.

Characteristics	Results
Age (years), mean (SD)	66.50 ± 11.82
Sex	
Male	15 (93.75%)
Female	1.00 (6.25%)
BMI (kg/m^2^), mean (SD)	24.5 ± 3.15
Disease course (years)	3.00 (1.00–7.00)
Duration of abrocitinib use (weeks)	12 (12–24)
Skin type	
III	13 (81.25%)
IV	3 (18.75%)
Phototest	
Only sensitive to UVA	7 (43.75%)
Only sensitive to UVB	1 (6.25%)
Sensitive to both UVA and UVB	8 (50%)
IgE (IU/mL)	693.65 (121.68–1168.75)

Data are present as median (inter-quartile range) or unless otherwise stated.

BMI, Body mass index.

Skin type, using the Fitzpatrick classification: type III sometimes mild burn and tan above average; type IV rarely cause burn, and tan more than average.

**Table 2:** Information of patients before and after treatment.

Patient no.	Sex/age, y	Disease course	Comorbidity	Skin type	UVA-MED	UVB-MED	Previous treatment	Concomitant medications	Duration of abrocitinib (weeks)	CAS-CAD scores at baseline	CAS-CAD scores at week 12
1	F/43	3	/	III	6.3	6.3	Antihistamine, topical corticosteroids	Antihistamine, topical corticosteroids	40	28.970	4.235
2	F/83	1	Hypertension	III	24.9	24.9	Antihistamine, topical corticosteroids	Antihistamine, topical calcineurin inhibitors	12	21.610	1.200
3	M/57	3	/	III	17.7	70	Antihistamine, topical corticosteroids	Antihistamine, topical corticosteroids, topical calcineurin inhibitors	24	17.230	4.330
4	M/65	4	/	IV	49.6	70	Cyclosporin A, hydroxychloroquine, antihistamine, topical corticosteroids	Antihistamine, topical calcineurin inhibitors	32	27.350	1.050
5	M/75	1	/	IV	49.6	49.6	Antihistamine	Antihistamine, topical corticosteroids, topical calcineurin inhibitors	12	12.760	0.410
6	M/78	1	Hypertension	III	8.9	8.9	Hydroxychloroquine, antihistamine, topical corticosteroids	Antihistamine, topical calcineurin inhibitors	12	22.945	1.645
7	M/61	3	Hypertension, chronic Type-B hepatitis	III	24.9	35.2	Hydroxychloroquine, antihistamine, topical corticosteroids	Antihistamine, antiviral therapy	24	20.690	0.950
8	M/58	10	/	III	24.9	49.6	Antihistamine, topical corticosteroids	Antihistamine, topical corticosteroids, topical calcineurin inhibitors	12	12.420	1.545
9	M/77	16	Hypertension, COPD	III	17.7	49.6	Antihistamine, Systemic corticosteroids, topical calcineurin inhibitors	Antihistamine, topical calcineurin inhibitors	20	25.660	2.105
10	M/66	7	Hypertension, psoriasis, chronic HBV carrier	III	24.9	35.2	Hydroxychloroquine, antihistamine, topical corticosteroids	Topical calcineurin inhibitors, antiviral therapy	12	14.300	0.790
11	M/69	0.5	Hypertension	III	24.9	49.6	Antihistamine	Antihistamine, topical corticosteroids	12	30.155	1.020
12	M/79	2	Hypertension	III	70	49.6	Dupilumab, Antihistamine, topical corticosteroids	Antihistamine, topical corticosteroids, topical calcineurin inhibitors	12	38.780	2.900
13	M/51	1	Hypertension, coronary heart disease latent tuberculosis	IV	49.6	70	Antihistamine, topical corticosteroids	Antihistamine, Antituberculosis drug, hydroxychloroquine, topical corticosteroids, topical calcineurin inhibitors	16	37.030	3.445
14	M/74	1	Hypercholesterolemia	III	8.9	12.5	Antihistamine, topical corticosteroids	Antihistamine, topical corticosteroids, topical calcineurin inhibitors	24	25.780	7.115
15	M/76	7	Atopic dermatitis	III	6.3	6.3	Antihistamine, topical corticosteroids, topical calcineurin inhibitors	Antihistamine, topical corticosteroids, topical calcineurin inhibitors	12	25.060	4.465
16	M/52	10	Latent tuberculosis	III	24.9	24.9	Antihistamine, topical corticosteroids	Antihistamine, Antituberculosis drug, topical corticosteroids	12	17.630	1.370

F, Female; M, Male; MED, Minimal erythema dose; COPD, Chronic obstructive pulmonary disease; HBV, Hepatitis B virus. Skin type, using the Fitzpatrick classification: Type III sometimes mild burn, tan about average, type IV rarely burn, and tan more than average. MED mean value: Type III: UVA-MED 38.5 J/cm^2^, UVB-MED 36.1 mJ/cm^2^; Type IV: UVA-MED 50.0 J/cm^2^, UVB-MED 47.0 mJ/cm^2^.

**Figure 1:**
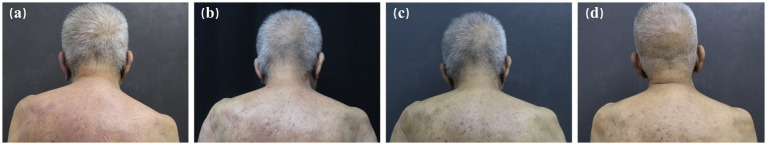
Clinical images of patient no. 9. **(a)** Before the treatment; **(b)** after 1 week of administration; **(c)** after 4 weeks; and **(d)** after 12 weeks.

**Figure 2:**
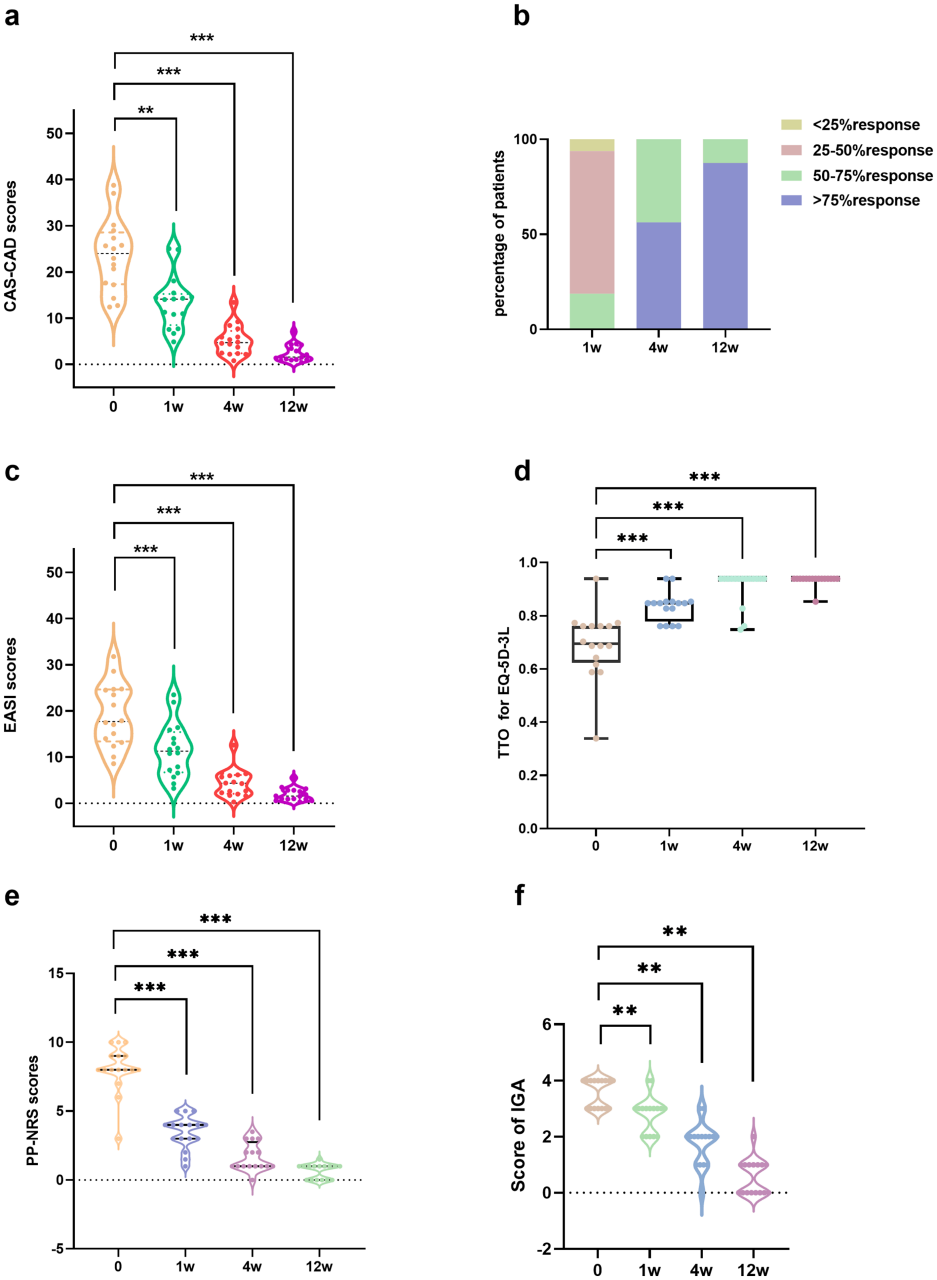
The changes of scores and clinical response before and after abrocitinib therapy. **(a)** The scores of CAS-CAD before and after abrocitinib therapy. **(b)** The achievement rate of excellent response, good response, partial response, and no response after 1, 4, and 12 weeks. **(c–f)** The scores of EASI, ED-5Q-3L, PP-NRS, and IGA before and after abrocitinib therapy. *p < 0.05, **p < 0.01, ***p < 0.001 compared with the respective value at baseline. CAS-CAD, Clinical active score of chronic actinic dermatitis; EASI, The eczema area and severity index; EQ-5D-3L, Three-level version of the EuroQol in five-dimension; IGA, Investigator’s global assessment; PP-NRS, Peak pruritus numeric rating scale itch; TTO, Time trade-off of values.

All 16 patients successfully completed the 12-week abrocitinib treatment at our hospital and underwent scheduled follow-up assessments. A significant reduction in skin lesions was observed after the treatment (Figure 1). At baseline, the CAS-CAD score of the patients was 23.65 ± 7.86. Following the intervention, CAS-CAD scores showed a progressive and significant reduction from baseline at 12 weeks of follow-up (2.41 ± 1.85, *p* < 0.001) (Figure 2a). The analysis of CAS-CAD scores shows that, during the first week 75% (12/16) of the cases achieved a partial response, three patients had a good response, and one patient received no response. At week 12, 87.5% (14/16) achieved excellent responses, and 12.5% (2/16) achieved a good response (Figure 2b). At all scheduled time points, CAS-CAD demonstrated good/excellent reliability with the ICC values for total score as follows: at baseline, ICC 0.949 (95% CI: 0.863–0.982); at 1 week, ICC 0.858 (95% CI: 0.646–0.948); at 4 weeks, ICC 0.835 (95% CI: 0.58–0.939); and at 12 weeks, ICC 0.859 (95% CI: 0.652–0.948). Future studies with larger sample sizes are expected to yield more precise estimates.

After the treatment of abrocitinib at weeks 1, 4, and 12, the scores of EASI, EQ-5D-3L, NRS, and IGA improved significantly compared with baseline levels (Figures 2c–f). At baseline, the patient’s EASI score was 19.06 ± 6.79. After 12 weeks, the EASI score showed a progressive and significant reduction compared to baseline (1.92 ± 1.40, *p* < 0.001). The EQ-5D-3L score for these patients was 0.69 ± 0.13 at baseline and 0.93 ± 0.02 after 12 weeks of treatment. While the NRS score was 8.06 ± 1.73, and the IGA score was 3.56 ± 0.51 at baseline, 0.66 ± 0.54 after 4 weeks, and 0.56 ± 0.63 after 12 weeks of treatment.

### 3.2 Safety of abrocitinib in treating CAD

In our enrolled patient cohort, all individuals were elderly, with 10 patients aged over 65 and 6 patients aged over 75. Notably, there is a paucity of data regarding individuals older than 75 in the dosage guidelines for abrocitinib. This study particularly highlights the need to consider safety aspects of treatment.

Among the 16 observed patients, one was a carrier of the hepatitis B virus, and another had active hepatitis B. With appropriate antiviral therapy concomitant with abrocitinib, no adverse events related to pathogen activation were observed. Two patients were identified with latent tuberculosis infection and were provided with single-agent preventive treatment with isoniazid for safety. No signs of active tuberculosis emerged during treatment. During follow-up, one individual experienced mild edema in both lower limbs after 24 weeks of abrocitinib use. A comprehensive evaluation, including laboratory tests and physical examination, suggested that the edema was unlikely to be related to cardiac disease, pulmonary edema, or deep vein thrombosis. Therefore, no additional interventions were initiated; abrocitinib was discontinued, and the edema resolved completely after drug withdrawal. Additionally, one patient developed folliculitis on the chest and back, which improved following symptomatic treatment. None of the remaining patients reported discomfort during treatment, and laboratory test results remained within normal ranges. For patients aged over 65 and over 75, laboratory tests were systematically monitored throughout the treatment course, and no adverse events such as hematological impairment, liver dysfunction, or renal damage were observed.

### 3.3 Recurrence of disease after reduction/discontinuation of abrocitinib

Following 12 weeks of observation, 9 patients initiated a dosage reduction regimen, taking 100 mg of abrocitinib once every other day, while a subset of patients (4 out of 16) opted to discontinue abrocitinib. They discontinued treatment primarily for socio-economic reasons rather than lack of efficacy or safety concerns. Among these individuals who stop abrocitinib (3 out of 4), new erythema and papules with itching developed, albeit with a lesser severity compared with pre-treatment levels. Remission was achieved with either through continued abrocitinib use or by transitioning to topical corticosteroids and antihistamines, which provided relief. In one case, a patient who had taken 100 mg of abrocitinib daily for 12 weeks experienced the reappearance of erythema and papules, along with worsened itching, upon reducing the dosage to 100 mg every 2 days, the symptoms subsequently subsided. This suggests that the initial duration of therapy may need to be extended in certain conditions, and the timing of dose reduction may require careful consideration.

### 3.4 Analysis of differentially expressed inflammation-related biomarkers

**Figure 3:**
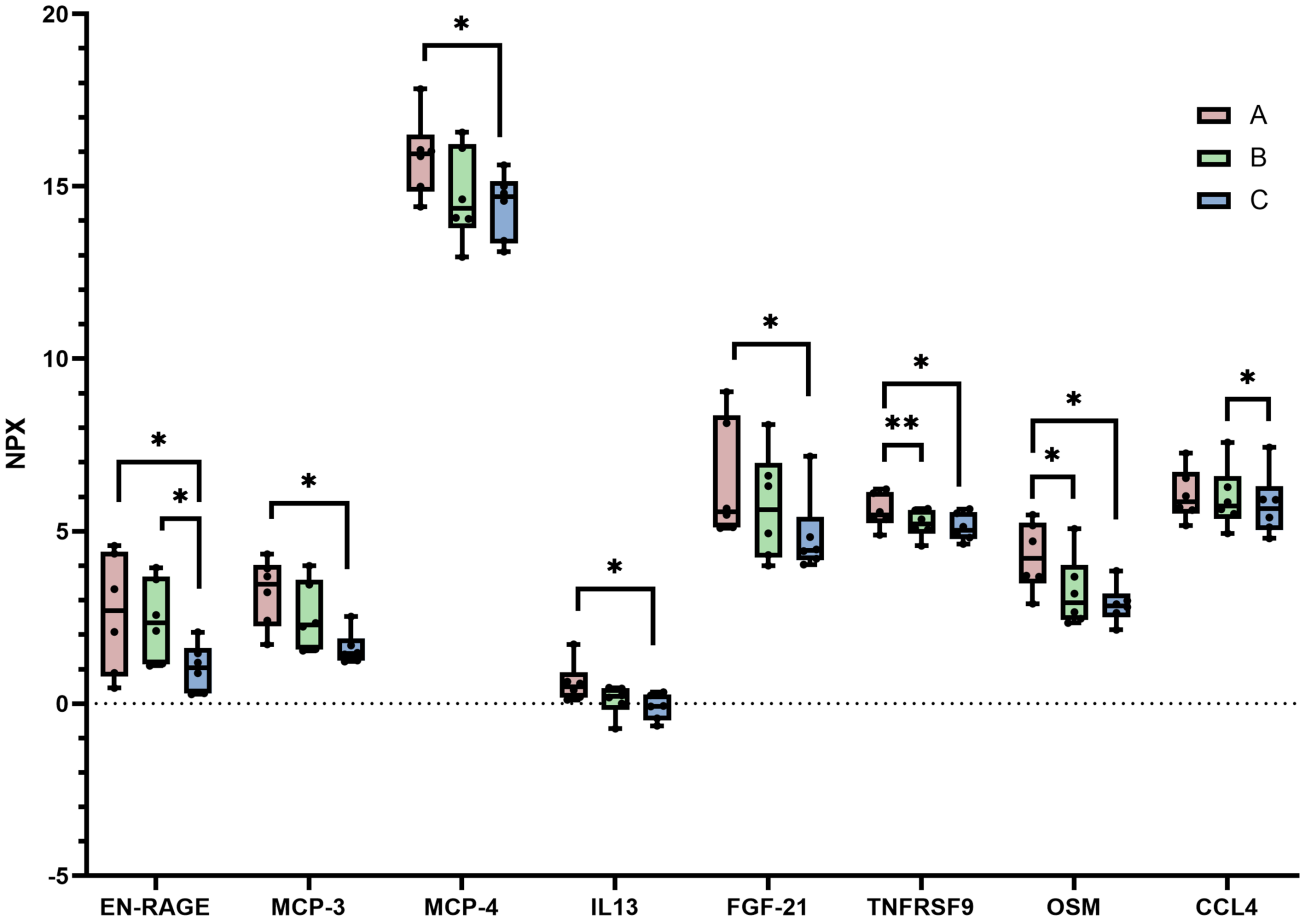
Protein expression before and after abrocitinib therapy. Seven differentially expressed proteins were identified between Group A and Group C: EN-RAGE, MCP-3, MCP-4, IL-13, FGF-21, TNFRSF9, and OSM, all of which were downregulated. Between Group A and Group B, two differentially expressed proteins were observed: OSM and TNFRSF9 were downregulated. Between Group B and Group C, two differentially expressed proteins were found: EN-RAGE and CCL4 were downregulated. Group A, baseline; Group B, after 1 week of abrocitinib therapy; and Group C, after 12 weeks of abrocitinib therapy. *p < 0.05, **p < 0.01.

We assessed plasma expression of 92 inflammatory proteins in patients at three time points—baseline (Group A), after 1 week of therapy (Group B), and after 12 weeks of therapy (Group C)—using Olink technology. Changes in specific proteins were observed over time. Notably, EN-RAGE, OSM, and TNFRSF9 levels gradually decreased with treatment duration compared with baseline. After 12 weeks, MCP-3, MCP-4, IL-13, and FGF-21 also exhibited significant reductions. CCL4 levels showed a significant decrease at 12 weeks post-treatment compared with levels observed at 1 week post-treatment (Figure 3).

**Figure 4:**
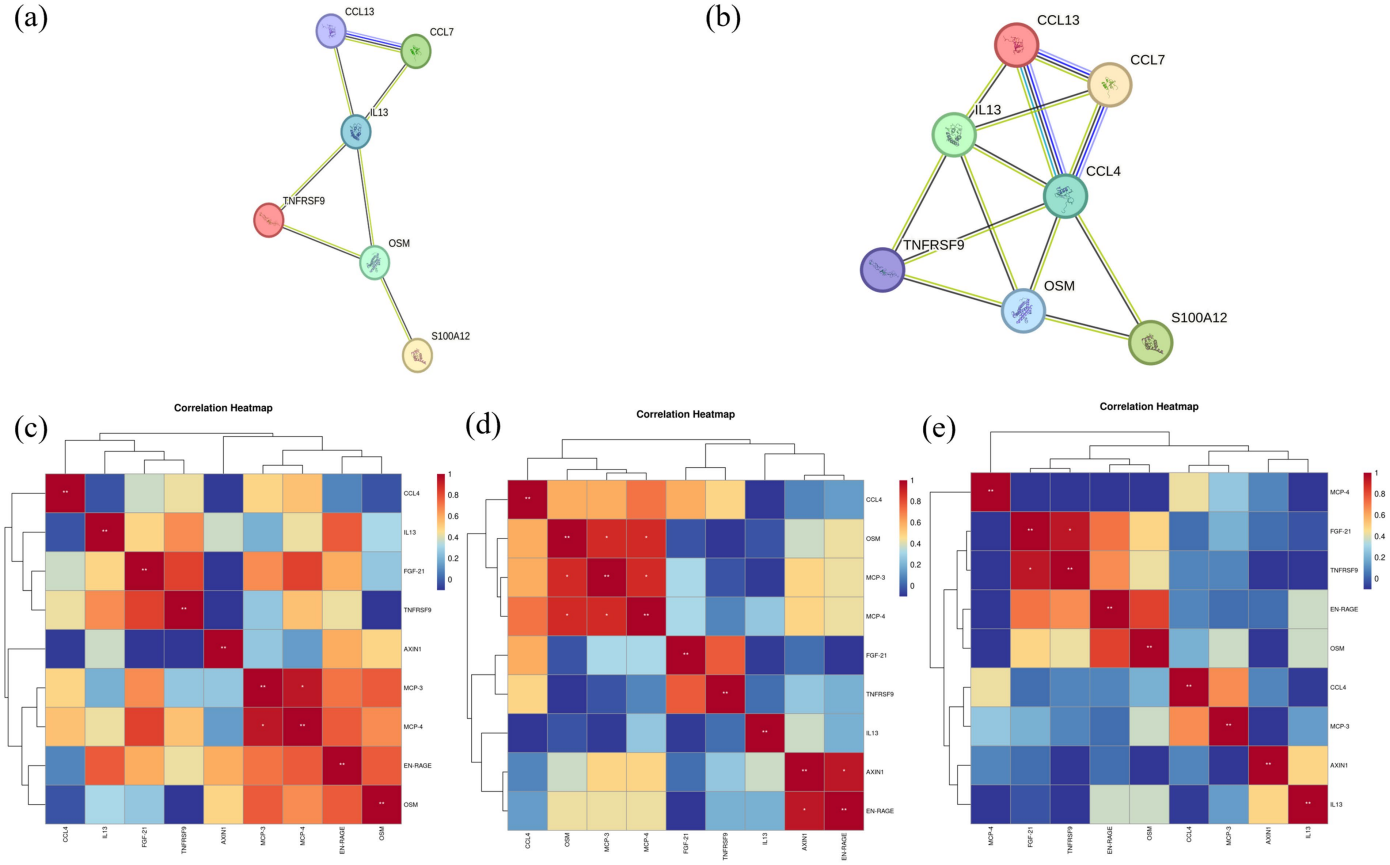
Analysis of differentially expressed proteins before and after abrocitinib therapy. Group A, baseline; Group B, after 1 week of therapy of abrocitinib; Group C, after 12 weeks of therapy of abrocitinib. **(a,b)** PPI network analysis between Group A and C, and among Group A, B, and C. **(c–e)** The correlation cluster marker heatmap illustrates the Spearman rank correlation between differentially expressed proteins (DEPs). Significance levels are denoted as follows: *p < 0.05, **p < 0.01, ***p < 0.0001. The normalized protein expression (NPX) values are used for the analysis. The color gradient represents the strength of the correlation, with red indicating a high positive correlation and blue indicating a low or negative correlation. Panels **(c–e)** display the correlation patterns at baseline, after 1 week of treatment, and after 12 weeks of treatment, respectively.

Using a paired *t*-test, we identified seven differentially expressed proteins between Groups A and C—EN-RAGE, MCP-3, MCP-4, IL-13, FGF21, TNFRSF9, and OSM—all down-regulated. The PPI network analysis highlighted IL-13 as a central protein in this set (Figure 4a). Between Groups A and B, OSM and TNFRSF9 were down-regulated; while between Groups B and C, EN-RAGE and CCL4 decreased. Across the three time points, eight proteins showed differential expression, with CCL4 and IL-13 identified as core proteins in the PPI network (Figure 4b). The changes observed in Figures 4a,b reflect alterations in inflammatory cytokines and the remodeling of inflammatory pathways in patients following abrocitinib therapy.

To gain deeper insights into the relationships between DEPs, we conducted correlation analysis visualized through heatmaps (Figures 4c–e). Particularly the core proteins CCL4 and IL-13 were focused. Before treatment, CCL4 exhibited moderate correlations with MCP-4, MCP-3, OSM, and FGF-21. Interestingly, these correlations intensified 1 week post-treatment, suggesting an early treatment effect on the inflammatory network. However, by 12 weeks, the strength of these correlations diminished significantly, indicating a potential stabilization or resolution of inflammation. Meanwhile, IL-13 initially showed strong correlations with EN-RAGE, TNFRSF9, FGF-21, and MCP-4. After 1 week of treatment, these correlations were notably reduced, implying a rapid impact on IL-13-associated pathways. This diminished correlation persisted through the 12-week treatment period, highlighting the sustained effect of abrocitinib in modulating IL-13-related inflammatory processes.

Given cytokine inter-correlations, pathway analysis provided more robust insights into effects of abrocitinib. GO and KEGG enrichment analyses showed that differential changes were largely associated with pathways in the negative regulation of cell proliferation, the positive regulation of inflammatory response, and inflammatory cell chemotaxis (Supplementary Figure 2, Supplementary Tables 1, 2), indicating specific molecular and biological functions potentially impacted by treatment.

## 4 Discussion

The precise pathogenesis of CAD remains a topic of ongoing investigation. Recent research has shed light on various contributing factors, including impaired skin barrier function, the involvement of IL-36γ, oxidative stress, and pyroptosis among others (3, 10–12) implies that CAD is a multifaceted condition influenced by multiple signaling pathways. Although there have been reports of promising results with the use of dupilumab in treatment of CAD in recent years (13), this monoclonal antibody primarily targets IL-4, and has certain limitations in terms of disease control. Instances of disease exacerbation post-dupilumab treatment have been documented (14). Thus, there is a need for exploration of new treatment modalities.

Inhibition of JAK results in broad-spectrum inhibition of cell signaling pathways including immune and epidermal cell-derived cytokines (e.g., thymic stromal lymphopoietin, IL-4, IL-13, IL-22, IL-31) (15). Upadacitinib has been proposed as a therapeutic agent for CAD (16). However, in the context of CAD, which predominantly affects elderly males, abrocitinib appears to be a more suitable choice due to its heightened selectivity for JAK1 and comparatively lesser inhibition of JAK3 (17). Whereas the inhibition of JAK3 is associated with CD8+ T-cell dysfunction and a decrease in the production of natural killer cells (18), abrocitinib may therefore have fewer adverse drug reactions (19).

When considering the use of JAK inhibitors, particular attention must be devoted to the potential side effects, particularly those related to opportunistic infections and impacts on the hematological system (20). In contrast to broad-spectrum JAK inhibitors, abrocitinib, functioning as a selective JAK1 inhibitor, offers superior safety profiles. However, abrocitinib is primarily recommended for patients between the ages of 12 and 75 years (21). In our study, we also gathered valuable insights from the usage of this drug in elderly patients aged over 75 years. Our study results reveal that the utilization of abrocitinib in CAD treatment boasts a commendable safety profile. Throughout the course of treatment, no disease exacerbation was observed following abrocitinib administration, and there were no instances of opportunistic infections. Over the 3-month treatment period, the drug did not induce severe hematological suppression. While one patient reported mild lower limb edema, clinical examinations did not confirm the occurrence of lower limb thrombosis, and the symptoms subsided upon discontinuation of the drug. Importantly, in elderly patients, the presence of hypertension and mild impairment in liver and kidney function did not significantly impede the administration of the drug.

The recurrence of skin lesions in CAD patients following abrocitinib treatment is attributed to several factors. Firstly, a rapid tapering or discontinuation of abrocitinib may contribute to recurrence. Abrocitinib is approved for treating AD in licensed doses of 100 mg and 200 mg, and the lower dose is recommended for adolescents as the starting dose for safety reasons (22). In our study, most of patients were elderly, so we also used 100 mg as the regular starting dose. Our dosage regimen involves administering abrocitinib at 100 mg daily for 12 weeks, followed by gradual tapering to 100 mg every 2 days over 8–12 weeks. If no new lesions or itching occur during tapering, further tapering to 50 mg every 2 days or discontinuation may be considered. Patient no. 1 in our observation had the longest duration, receiving abrocitinib at 50 mg every 2 days without recurrence or worsening of pruritus. Secondly, non-standardized medication and lax sun protection contribute to recurrence. Some patients irregularly take abrocitinib after completing the 12-week course. Some adopt an on-demand approach, taking the medication for 1–2 weeks when itching intensifies and discontinuing when it eases. Others take 100 mg every 2–3 days. Patients with irregular recurrence commonly exhibit lax sun protection practices as they have poor compliance. Notably, visible light emitted by indoor incandescent lamps also exacerbate CAD (23), emphasizing the significance of patient education and regular follow-up. Additionally, some patients continue to consume photosensitive foods, which is also detrimental to disease control.

In our study, we observed that one patient, who was treated as planned, experienced a relapse during dose tapering. This suggests that the timing of rapid induction of remission and dose reduction for maintaining abrocitinib efficacy in CAD treatment requires careful consideration. A study showed that the majority of AD patients who received an induction-maintenance approach with reduced-dose abrocitinib were relapse-free for at least 40 weeks, while reporting fewer treatment-emergent adverse events (TEAES) (24). This suggests that a combination of cost/risk benefit needs to be considered when determining the optimal maintenance dose for an individual patient, which is also a focus that must be clarified for our subsequent study.

One patient presented with clinical features of two concurrent dermatological conditions. Scaly plaques with positive “wax drop” and Auspitz signs were observed on the limbs and back, and a skin biopsy confirmed the diagnosis of psoriasis. Erythema and lichenified changes were noted on the forehead and posterior neck, with histopathological findings consistent with CAD. The patient had a history of coronary heart disease and was receiving metoprolol. As *β*-blockers have been reported to induce psoriasis (25), a cardiology consultation was obtained, and metoprolol was replaced with ivabradine, while abrocitinib therapy was also initiated. Following treatment, improvement was observed in both dermatoses. This suggests that abrocitinib may also benefit patients with CAD who present with coexisting inflammatory skin conditions.

In this study, we used Olink proteomic analysis of the inflammation panel to study the protein changes in CAD patients before and after abrocitinib therapy, and the results showed significant changes in several inflammatory factors such as EN-RAGE, MCP-3, MCP-4, IL-13, FGF21, and TNFRSF9. The PPI network suggested that IL-13 and CCL4 might be the core proteins. The receptor for RAGE (advanced glycation end products) is a versatile receptor with multiple ligands, including advanced glycation end products, high mobility group box protein 1, S100/calgranulin, beta-amyloid, phosphatidylserine, C3a, and advanced oxidation protein products (26). These ligands, produced by various inflammatory cells, initiate signal transduction that influences numerous pro-inflammatory pathways, perpetuating a chronic inflammatory state. In our study, reduced RAGE levels with treatment suggest a shift toward a less inflammatory state. IL-13, secreted by Th2 lymphocytes and type 2 innate lymphocytes (ILC2), plays a crucial role in AD by impairing epidermal barrier function and promoting inflammation (27). The observed decrease in IL-13 expression levels aligns with the therapeutic effect in CAD, indicating a potential reduction in inflammatory drivers. Similarly, CCL4, an essential chemokine in immune cell recruitment and inflammatory response (28), also decreased significantly post-treatment, further supporting clinical improvement in CAD. TNFRSF9, a critical costimulatory molecule within the TNFRSF family, influences T cell proliferation, survival, and differentiation (29). Its differential expression may reflect an underlying negative regulatory mechanism within the inflammatory cascade of CAD, potentially contributing to the down-regulation of chronic inflammation. A reduction in monocyte chemoattractant protein (MCP) expression was also observed in this study. MCP is well known for inducing monocyte/macrophage migration (30), and the dermatopathology of CAD patients shows a lymphocyte-dominated infiltration (2), which suggests that MCP may also be involved in the genesis and progression of CAD. These bioinformatic results indicated that inflammation may be involved in the pathogenesis of CAD through a multifaceted mechanism. Further studies are needed to explore the role of these signaling pathways in the development of CAD.

In summary, our findings indicate that abrocitinib demonstrates efficacy in CAD treatment, presenting a promising therapeutic alternative for patients averse to corticosteroid or injectable treatments. Additionally, our observations of changes in specific protein levels before and after treatment may provide useful information for future research on the mechanisms of CAD. This exploratory study was primarily designed to conduct an initial assessment of the efficacy and safety of abrocitinib for CAD, thereby establishing a clinical foundation and a framework for further investigation. However, this study has certain limitations, including a limited sample size, potential biases, and lack of ethnic diversity in the study population. Therefore, larger-scale, multicenter, randomized controlled trials are needed to validate these findings. Moreover, as only the 100 mg QD dosing regimen of abrocitinib was examined, optimized dosing strategies remain to be explored.

## Data Availability

The datasets presented in this study can be found in online repositories. The names of the repository/repositories and accession number(s) can be found at: https://figshare.com/, 10.6084/m9.figshare.30571460.

## Supplementary material

SUPPLEMENTARY FIGURE 1 Clinical active score of chronic actinic dermatitis scoring calculation details. **(a)** Classification based on dressing habits. **(b)** Calculation of the affected area relative to body surface area. **(c)** Final score calculation based on the severity of different types of skin lesions.

SUPPLEMENTARY FIGURE 2 Gene Ontology (GO) terms and Kyoto Encyclopedia of Genes and Genomes(KEGG) of the significant inflammation proteins. Group A, baseline; Group B, after 1 week of therapy of abrocitinib; Group C, after 12 weeks of therapy of abrocitinib. (a–c) GO enrichment analyses of differentially expressed proteins:(a) Group A vs. Group B;(b) Group A vs. Group C;(c) Group B vs. Group C. (d–f) KEGG pathway enrichment analyses of differentially expressed proteins:(d) Group A vs. Group B;(e) Group A vs. Group C;(f) Group B vs. Group C.
