# Supplementary figures and images for: Extended treatment of abrocitinib: evaluation of efficacy and safety in chronic actinic dermatitis
Source: Front Med (Lausanne). 2026 Feb 4;13:1742273. doi: 10.3389/fmed.2026.1742273 (PMC12913078; doi:10.3389/fmed.2026.1742273)

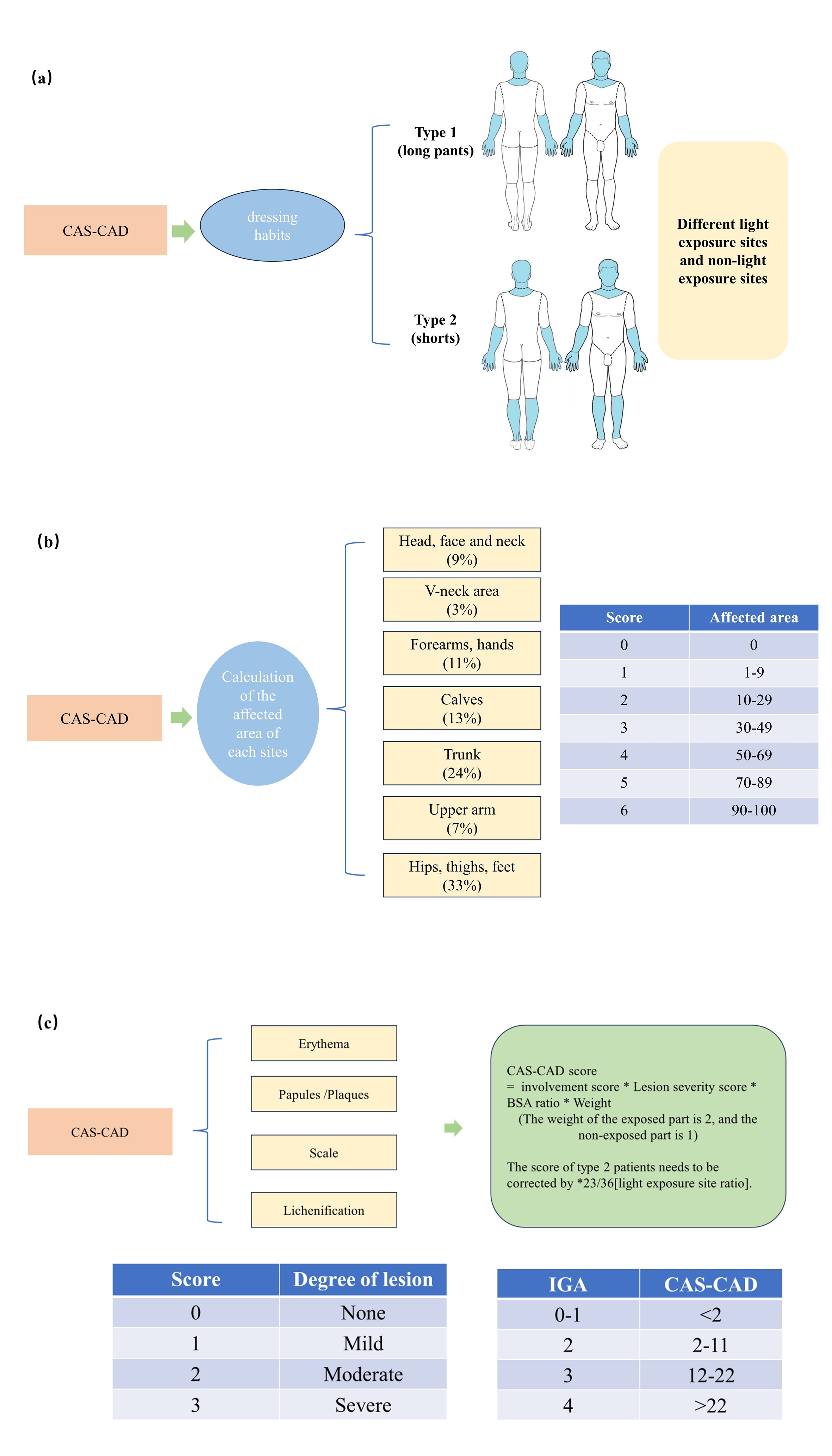

Supplement: SUPPLEMENTARY FIGURE 1 — Clinical active score of chronic actinic dermatitis scoring calculation details. (a) Classification based on dressing habits. (b) Calculation of the affected area relative to body surface area. (c) Final score calculation based on the severity of different types of skin lesions. [file Image_1.JPEG]

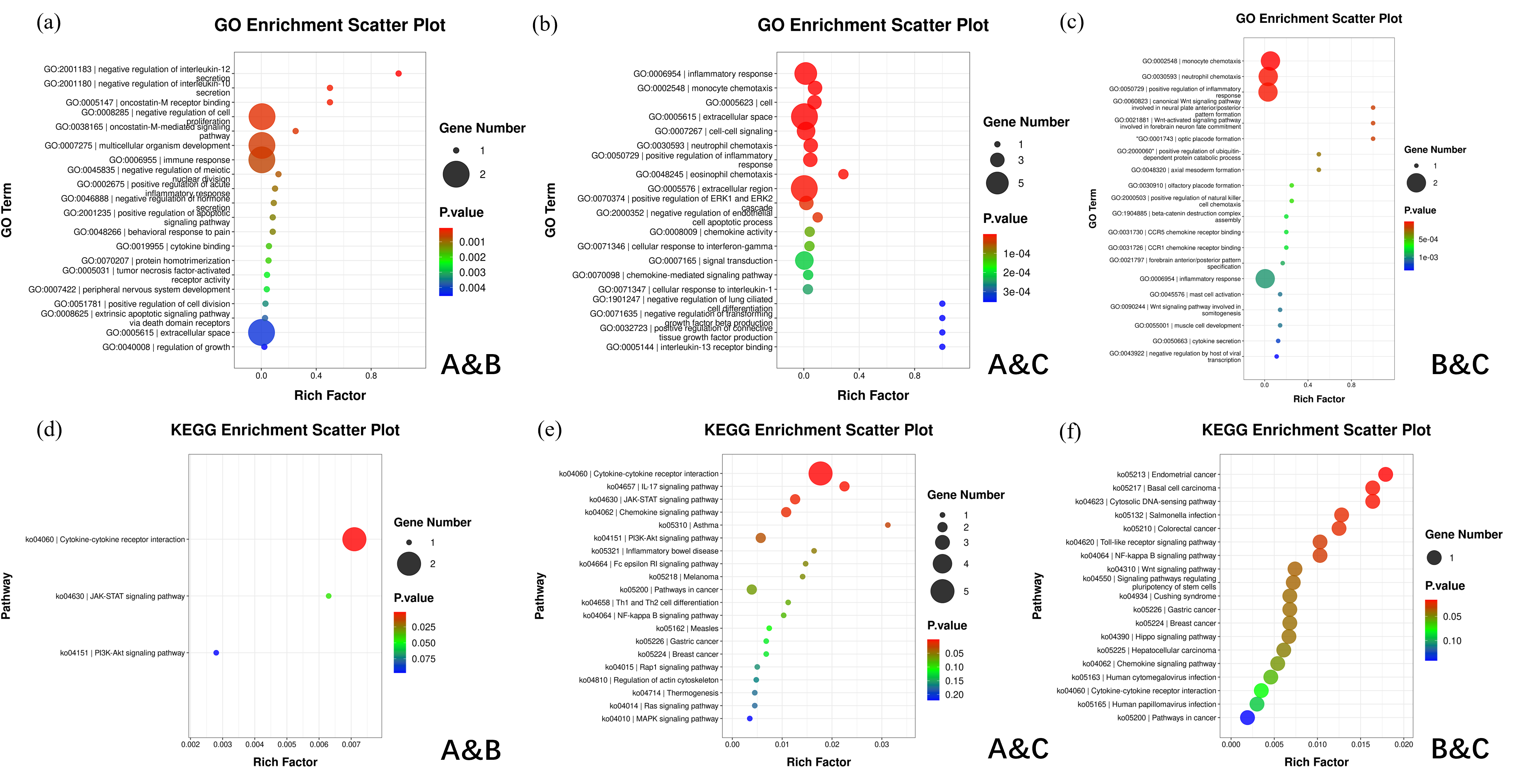

Supplement: SUPPLEMENTARY FIGURE 2 — Gene Ontology (GO) terms and Kyoto Encyclopedia of Genes and Genomes(KEGG) of the significant inflammation proteins. Group A, baseline; Group B, after 1 week of therapy of abrocitinib; Group C, after 12 weeks of therapy of abrocitinib. (a–c) GO enrichment analyses of differentially expressed proteins:(a) Group A vs. Group B;(b) Group A vs. Group C;(c) Group B vs. Group C. (d–f) KEGG pathway enrichment analyses of differentially expressed proteins:(d) Group A vs. Group B;(e) Group A vs. Group C;(f) Group B vs. Group C. [file Image_2.TIF]
