# Supplementary material for: Extended treatment of abrocitinib: evaluation of efficacy and safety in chronic actinic dermatitis
Source: Front Med (Lausanne). 2026 Feb 4;13:1742273. doi: 10.3389/fmed.2026.1742273 (PMC12913078; doi:10.3389/fmed.2026.1742273)
Supplement: Supplementary file 3 [file Table_1.DOCX]

| **Supplemental Table 1 GO pathway enrichment analysis of DEP**s | | |
| --- | --- | --- |
| Term | Count | P-value |
| GO:0006954 inflammatory response | 5 | 3.86E-08 |
| GO:0002548 monocyte chemotaxisv | 3 | 2.10E-07 |
| GO:0005623 cell | 3 | 2.47E-07 |
| GO:0005615 extracellular space | 6 | 4.93E-07 |
| GO:0007267 cell-cell signaling | 4 | 7.82E-07 |
| GO:0030593 neutrophil chemotaxisv | 3 | 9.22E-07 |
| GO:0050729 positive regulation of inflammatory response | 3 | 1.12168E-06 |
| GO:0048245 eosinophil chemotaxis | 2 | 2.25E-06 |
| GO:0005576 extracellular region | 6 | 3.52E-06 |
| GO:0070374 positive regulation of ERK1 and ERK2 cascade | 3 | 1.91378E-05 |
| GO:2000352 negative regulation of endothelial cell apoptotic process | 2 | 2.03519E-05 |
| GO:0008009 chemokine activity | 2 | 0.000115265 |
| GO:0071346 cellular response to interferon-gamma | 2 | 0.000125353 |
| GO:0007165 signal transduction | 4 | 0.000212471 |
| GO:0070098 chemokine-mediated signaling pathway | 2 | 0.000214347 |
| GO:0071347 cellular response to interleukin-1 | 2 | 0.00024204 |
| GO:0005144 interleukin-13 receptor binding | 1 | 0.000354036 |
