# Supplementary material for: Extended treatment of abrocitinib: evaluation of efficacy and safety in chronic actinic dermatitis
Source: Front Med (Lausanne). 2026 Feb 4;13:1742273. doi: 10.3389/fmed.2026.1742273 (PMC12913078; doi:10.3389/fmed.2026.1742273)
Supplement: Supplementary file 4 [file Table_2.DOCX]

| **Supplemental Table 2 KEGG pathway enrichment analysis of DEPs** | | |
| --- | --- | --- |
| Term | Count | P-value |
| ko04060: Cytokine-cytokine receptor interaction | 5 | 5.90E-07 |
| ko04657: IL-17 signaling pathway | 2 | 0.002296205 |
| ko04630: JAK-STAT signaling pathway | 2 | 0.007171041 |
| ko04062: Chemokine signaling pathway | 2 | 0.009721245 |
| ko05310: Asthma | 1 | 0.02698932 |
| ko04151: PI3K-Akt signaling pathway | 2 | 0.03256993 |
